# 'To take care of the patients': Qualitative analysis of Veterans Health Administration personnel experiences with a clinical informatics system

**DOI:** 10.1186/1748-5908-5-63

**Published:** 2010-08-20

**Authors:** Laura M Bonner, Carol E Simons, Louise E Parker, Elizabeth M Yano, JoAnn E Kirchner

**Affiliations:** 1 Health Services Research and Development Northwest Center of Excellence for Outcomes Research in Older Adults, VA Puget Sound Healthcare System, Seattle, WA, USA; 2 Department of Psychiatry & Behavioral Sciences, University of Washington School of Medicine, Seattle, WA, USA; 3 Independent Consultant, Cambridge, MA, USA; 4 Health Services Research and Development Center for Mental Healthcare and Outcomes Research, North Little Rock, AR, USA; 5 Health Services Research and Development Center of Excellence for the Study of Health Care Provider Behavior, VA Greater Los Angeles Healthcare System, Sepulveda, CA, USA; 6 School of Public Health, University of California, Los Angeles, CA, USA; 7 VA South Central Mental Illness Research, Education, and Clinical Center, Central Arkansas Veterans Healthcare System, North Little Rock, AR, USA; 8 University of Arkansas for Medical Sciences, Little Rock, AR, USA; 9 Health Services Research and Development Center for Mental Healthcare and Outcomes Research, Central Arkansas Veterans Healthcare System, North Little Rock, AR, USA

## Abstract

**Background:**

The Veterans Health Administration (VA) has invested significant resources in designing and implementing a comprehensive electronic health record (EHR) that supports clinical priorities. EHRs in general have been difficult to implement, with unclear cost-effectiveness. We describe VA clinical personnel interactions with and evaluations of the EHR.

**Methods:**

As part of an evaluation of a quality improvement initiative, we interviewed 72 VA clinicians and managers using a semi-structured interview format. We conducted a qualitative analysis of interview transcripts, examining themes relating to participants' interactions with and evaluations of the VA EHR.

**Results:**

Participants described their perceptions of the positive and negative effects of the EHR on their clinical workflow. Although they appreciated the speed and ease of documentation that the EHR afforded, they were concerned about the time cost of using the technology and the technology's potential for detracting from interpersonal interactions.

**Conclusions:**

VA personnel value EHRs' contributions to supporting communication, education, and documentation. However, participants are concerned about EHRs' potential interference with other important aspects of healthcare, such as time for clinical care and interpersonal communication with patients and colleagues. We propose that initial implementation of an EHR is one step in an iterative process of ongoing quality improvement.

## Background

Recent research and national healthcare policy discussions have highlighted the potential of electronic health records (EHRs) to improve quality and efficiency [1-3] and potentially to reduce healthcare costs [4,5]. Many large healthcare organizations have implemented some form of healthcare informatics, but few have comprehensive systems [6]. EHRs have been difficult to implement [7], and their cost-effectiveness remains unclear [8-10]. For example, the British National Health Service has experienced 'costly delays' in implementation of its EHR [11]. Researchers have identified many barriers to implementation, including increased documentation time [12,13], interference with clinical workflow, apprehension about unintended negative consequences, financial concerns, physician resistance, maintenance costs, and inadequate information technology (IT) staff to support implementation, among others [6,14,15].

The Plan-Do-Study-Act cycle (PDSA) provides a useful framework for evaluating system change [16], and can be used to conceptualize EHR implementation. Informatics systems such as the EHR are designed and built to meet clinical needs (in the 'Plan' phase). The EHR is then implemented (in the 'Do' phase), and end-users provide feedback (during the 'Study' phase) that drives further refinement of the informatics system (during the 'Act' phase). In this framework, feedback from end-users is essential to make the EHR more acceptable to clinicians and more useful to the organization. The ITSA model [17] likewise describes a recursive relationship in which interactions between health IT and the larger clinical environment shape development of both the EHR and the larger environment. In both models, awareness of how end-users interact with the EHR is essential for successful implementation and improvement of the informatics system. Concretely, structured usability testing can generate valuable data about what end-users like and dislike about software. Likewise, in an article describing the implementation of the Veterans Health Administration's (VA) EHR, Evans and colleagues identify an 'iterative partnership' between users and developers as central to the success of EHR implementation [18].

The VA has invested significant time and resources in the development and implementation of a sophisticated, multifunctional EHR [19]. The VA first implemented its EHR, the Computerized Patient Record System (CPRS), widely in the mid-1990's, and today there is almost universal CPRS use among VA clinicians [19]. Among other important functions, CPRS supports communication among treatment team members and provides decision support in various forms, including reminders for important clinical tasks [20].

The purpose of this article is to describe VA staff members' experiences with the VA's EHR, as implemented in clinical settings. Participants describe both barriers to implementation and the value added to the organization by the EHR. Participants' recommendations may help healthcare administrators anticipate barriers to EHR implementation and work to address them, while at the same time increasing adoption by enhancing the features valued by staff.

## Methods

We collected the data presented here as part of the Cost and Value of Evidence-Based Solutions for Depression Study (COVES) [21,22]. COVES evaluated the VA TIDES [23,24] (Translating Initiatives for Depression into Effective Solutions) depression care initiative, a clinic-level quality improvement (QI) intervention to enhance depression treatment in primary care. The VA is a national healthcare system, divided into 21 distinct geographic regions or VISNs (VA Integrated Service Networks). The TIDES team implemented the program in seven primary care clinics across three VISNs.

As part of the COVES study, pairs of investigators conducted semi-structured interviews with VA personnel at five of the seven participating TIDES sites. We were unable to conduct interviews at one of the sites because the site experienced extremely severe hurricane damage. One other site was one of two clinics affiliated with the same parent facility; interviews were conducted at the other of those clinics. The study received Institutional Review Board (IRB) review and approval from participating institutions as well as from the administrative sites. We conducted the majority of the interviews (N = 67) in face-to-face meetings during site visits; we conducted telephone interviews with five additional participants who were not available during our site visits for a total of 72 interviews with VA personnel. We provide a description of participants' organizational roles (Table 1). At each site, we selected participants who had been exposed to the TIDES intervention and who represented different disciplines and different positions within the organization. Our goal was to gain a wide representation of VA stakeholders rather than a complete set of stakeholders from one site or discipline. We believe this sampling strategy accurately reflects the real-world implementation process, in which the success of a given initiative depends on support across sites and disciplines.

**Table 1:** Participant Characteristics

Participant Role	Number of participants
Primary Care Physician	18

Primary Care Physician Assistant	5

Primary Care Advanced Practice Nurse (APN)	1

Primary Care Registered Nurse (RN)	10

Psychiatrist	5

Mental Health APN	3

Psychologist	1

Mental Health Social Worker	1

Non-clinical administrator	5

Medical center or regional network manager	19

Care managers (RNs specializing in depression disease management)	4

A psychiatrist, a psychologist, a social worker, and two doctoral level health services researchers served as interviewers. We audio-recorded all interviews and analyzed the resulting verbatim transcripts using qualitative data management software [25]. The research team developed 22 top-level codes relating to different aspects of the TIDES intervention. Four investigators (the first, second, and third authors and one of the interviewers) conducted the top-level coding, assigning codes to blocks of text (*i.e.*, quotations) within transcripts. Quotations are frequently associated with multiple codes. After the initial top-level coding process, two investigators reviewed 20% of the interview transcripts for coding consistency. The methodology for ensuring coding consistency has been described in detail elsewhere [21]. Coding agreement statistics were not calculated. Rather, these two investigators worked with other investigators to reach coding consistency, resolved disagreements through discussion and consensus, and reviewed codes that investigators had difficulty coding consistently. Also, word searches were conducted on all transcripts to detect any missing code-content links. For example, the word 'email' was searched to ensure that all quotations containing 'email' were properly coded with the 'informatics' code (as well as any other applicable codes). This intensive process ensured a high degree of coding consistency.

We created 22 top-level codes for this study. In this article, we present data relating to one of these codes, 'informatics'. We list the other 21 codes in Table 2. These codes either reflect other aspects of the implementation process or are specific to the parent QI initiative. The study was very large, yielding thousands of pages of qualitative data. It would not be possible to integrate all of these data into one meaningful paper. Articles integrating several other codes have been published or are currently in preparation. One published article [21] integrates subcodes of the following top-level codes: 'implementation/spread process', 'participation in design and customization' and 'ideal model' to describe the process of quality improvement within healthcare organizations. In a manuscript in press, Kirchner and colleagues analyze subcodes of the 'implementation/spread process' and 'ideal model' codes specifically in relationship to different stakeholders' perspectives; they have presented this work at a conference [22]. Parker and colleagues synthesized information from the 'clinical innovativeness' and 'individual, site, VAMC, VISN, and VA characteristics' codes for a conference presentation [26] and are currently preparing a related manuscript. Yano and colleagues are currently preparing a methodological manuscript integrating six subcodes of the 'TIDES activities' code to describe our approach to measuring implementation fidelity, in this case fidelity to the original depression collaborative care model elements. The 'TIDES program ranking rationale' and 'DCM ranking rationale' codes formed the basis of a conference presentation [27]. Some codes, including 'human subjects' activities', yielded relatively little information, and we are therefore unlikely to develop papers based on them.

**Table 2:** Top-level codes

TIDES Activities
Implementation/Spread Process

Involvement

Participation in Design and Customization

Barriers to quality depression care

TIDES Positive

TIDES Negative

Change in attitudes and behavior since TIDES

Remain post-study

TIDES program ranking rationale

DCM ranking rationale

Depression as a chronic illness

Facility depression care quality

Ideal model/suggestions for improvement

TIDES model population applicability

Clinic interaction/collaboration

Clinic innovativeness

Individual, site, VAMC, VISN & VA Characteristics

Informatics

Perceived consumer ability to affect change

Consumer depression related interest and activity

Human subjects issues

The themes derived from the 432 informatics quotations did not generally integrate well with findings from the other codes, and thus would not have been appropriate for incorporation into other publications (although some individual quotations were assigned a code or codes in addition to 'informatics'). Respondents made comments about the VA EHR in general; they did not confine their remarks to the role of the EHR in this QI project. Therefore, we have chosen to present 'informatics' separately from other codes. Codes were not separated by site, or by profession of respondent. However, we note the respondent's profession with each quote as this information may provide important context for the reader.

Two investigators (the first and second authors) developed sub-codes that reflected the content of quotations associated with the informatics top-level code (see Table 3). The same two investigators each worked with one-half of the transcripts and assigned one or more subcodes to all quotations associated with the informatics top-level code. These two investigators then reviewed each other's sub-coding and met to resolve discrepancies. Finally, these investigators developed summaries of the themes discussed in relation to each subcode.

**Table 3:** Subcodes and number of associated quotations

Informatics sub-codes	Number of associated quotations
'Informatics' top-level code	432*

Electronic communication and connectivity/telemedicine	181

Utilization or lack of utilization of informatics system by providers and patients	161

Decision support	131

Collaborative care and informatics	129

Health information and data	126

Positives	98

Negatives	68

Barriers to informatics system implementation or use	61

Suggestions/improvements	58

Website/internet use	36

Marketing of informatics system/training	28

Reporting and population health management	22

PHQ9/other instruments	19

Usability	18

Patient support	12

IT support required	9

Administrative process	7

Order entry/order management	6

Cost of informatics	3

Results management	1

*Note that some quotations were assigned more than one subcode.

## Results

### Barriers

Study participants described barriers to their use of the EHR. Recent research documents that the average primary care visit takes 20.8 minutes, with additional time required for counseling and screening [28]; other research has found that about five minutes are allocated to the longest topic during the visit, with each additional topic receiving slightly more than one minute [29]. Time has been identified as a significant barrier to use of clinical reminders [30]. Participants accordingly expressed concerns about time management:

'CPRS is great, but it takes time to use ... [Providers] have to see very complicated patients in 20 minutes, and so anything that's in addition to is going to be negatively perceived ... With every point and click on a computer it's less time they spend with a patient. They generally just want to take care of the patients.' (Primary Care Nurse)

'I'm the click counter. I think one time I sent [an administrator] an e-mail about how many clicks it took to take care of a diabetic patient, because I clicked through all the reminders and I mean it's hundreds.' (Primary Care Physician)

As converging evidence, barriers to the effective use of clinical reminders have been documented previously. These barriers include number of reminders and presentation of inapplicable reminders [30].

Another barrier was apprehension that the EHR would lead to impersonal interactions between staff and patients, and perhaps even between staff members. Clinicians expressed concerns about the impersonal nature of reminder-driven interactions, which in their experience made filling out forms rather than listening to patients the priority:

'Well, you know, clinical reminders are fine, but less and less they bring in independent thought, a provider that asks the right questions and show interest in the patient.' (Psychiatrist)

'I just feel like that the personal ... I mean, what happened with talking face to face with someone.' (Primary Care Physician Assistant)

'All these blasted checklists, clerks should be doing that.... Doctors need to sit there and look someone in the eye ... What's really bothering you? How can I help you today?' (Psychiatrist)

As converging evidence, DeBlasio and Walker [31] examined the perceived quality of care delivered in a simulated medical interview. Simulated interviews using a desktop computer were rated lower than those using less obtrusive technologies or no technology, suggesting that EHR use may be perceived as interfering in the clinical relationship.

Complex clinical discussions require interpersonal trust between professionals, and it is preferable to conduct sensitive discussions in person. In the words of one case manager, 'I'm asking physicians in [another VA facility] to know me and trust me simply by what they have read in my progress notes ... and most of them have not met me personally.'

A primary care RN observed, '[T]here are a lot of things you don't want to put it as a formal note in the patient chart.' Likewise, a case manager describes the problems that arise when clinicians use the chart for clinical conversations:

'There have been a couple of times where I've found that the providers will respond back to me as if they're forgetting that they're in a patient's medical record and will say what would you like me to do where that's not ... appropriate.'

### Values

Study participants described the value added by specific functions of the EHR, including notes used for communication and structured consults used to increase efficiency and educate providers. Participants used the electronic medical chart itself, not a separate email function, to support an asynchronous, secure conversation about treatment decisions. Participants used the cosign function, which enables one clinician to generate a note, and then name another as a cosigner, as a useful way of bringing matters to the correct person's attention and asking for the recipient's feedback, which was easily provided as an addendum to the original note. A psychologist mentioned the value of such conversations in supporting interdisciplinary collaboration: '[O]ur computerized record system ... makes it awfully easy for the mental health, primary care to work with the other on what's going on.'

Some participating clinics had recently implemented a depression clinical reminder when we conducted this study. Clinical reminders about required screenings and other tasks initially appear in the EHR when a patient arrives in the clinic and a nurse administers an initial screening. When the primary care provider opens the patient's EHR, the results of the screening are available, and the provider follows up as clinically indicated. These structured screenings add value by opening up important provider-patient discussions:

'[S]eeing so many patients a day, [the clinical reminder] reminds us to talk with these people and ask these patients ... are you feeling depressed ... if we didn't have the reminders, we may not take the time to do that.' (Primary Care Nurse)

Clinical reminders support a structured conversation with patients about potentially sensitive topics, in this case depression. Some participants appreciated the role of the clinical reminders in facilitating personal interaction between professional and patient:

'I feel that we probably because we took the time to really spend with them ... asking them questions, I really feel that we got a lot of people to talk to us about their depression ...' (Primary Care Registered Nurse)

Based upon all of our data, it is not possible to determine whether a majority of respondents liked or disliked clinical reminders. It is more accurate to state that respondents saw both positives and negatives of reminders, likely due to many variables that we did not capture, such as respondent profession, differences in the number of reminders presented, and other factors.

Another form of decision support is a structured consult form that provides the referring clinician with specific guidance about which clinical variables to assess, which interventions to begin and what information to include in referrals. Several participants valued the ability of structured consults to educate providers about best practices:

'We make it an effort to try to educate our colleagues by essentially templating the consults so it requires them to answer those questions that we need....' (Psychiatrist)

'[Y]ou can have a consult form that asks questions or builds in information ... and has force fields so you say ... here are the diagnostic criteria, here are the screening criteria, has your patient met these?... Have you done this kind of assessment?... Do they have contraindications? Have you tried this initial intervention?' (Primary Care Physician Administrator)

Although, as discussed above, time management concerns constituted a barrier to informatics use, some participants valued the time efficiency of asynchronous communication and rapid referrals provided by the EHR. A physician administrator said, 'I thought [the clinical reminder] was really slick ... with the click of the button you could refer them.' Likewise, a nurse care manager stated, 'A lot of the time, you know, you can stand outside the door and wait, and then they're busy throughout the day, and CPRS, you know, they can get to it whenever they have time for it.'

Finally, participants discussed EHR implementation in the context of the larger healthcare system. It is important to design the system of care so that implementation of informatics promotes good clinical practice. The following statements express the importance of organizational context:

'We don't flunk in depression screening, in catching it, we flunk in follow up of the depression screening.' (Primary Care Physician)

'[I]f the providers are overwhelmed with clinical reminders, they become somewhat numb to them ... It's also a system issue.' (Psychologist)

## Discussion

VA personnel described complex perceptions of the EHR. Rather than providing a simple list of barriers, respondents discussed the advantages and disadvantages they perceived in the EHR. For example, respondents described the efficiency and convenience of the EHR, but also acknowledged that such convenience could encourage documentation of informal remarks that are not appropriate in the patient's record.

Respondents revealed two important barriers to EHR implementation: concerns about the technology taking time away from patient care and apprehension about the technology detracting from interpersonal relationships (refer to Table 4 for a summary of barriers and valued aspects of the EHR). These barriers are consistent with published reports of providers' and patients' concerns about EHRs [31-34]. Awareness of these barriers suggests solutions for future implementation efforts. For example, future informatics design could minimize the data entry time required of clinicians. Thought could be given to incorporating less intrusive technologies, rather than desktop computers, where possible in the clinical interaction, or re-positioning computers to maximize face-to-face discussion. Clinicians may also consider spending a brief time talking with patients without using the EHR in order to build rapport. Secure messaging, possibly separate from the official medical record, might facilitate clinical consultation.

**Table 4:** Values of and barriers to EHR use

Valued attributes and functions of the EHR	Barriers and concerns about use of the EHR
Time: Asynchronous communication allows VA personnel to send and receive information at a time convenient for them	Time: time required to complete reminders

Documentation: Support for appropriate documentation	Impersonality: with colleagues--inappropriate conversations becoming part of medical record

Communication: Can easily alert other providers about a patient's status	Impersonality: with colleagues--trust

Quality of care: Reminders prompt providers to initiate important conversations	Impersonality: with patients

Quality of care: Structured consults and reminders provide guidance to providers about evidence-based priorities	Systems issues: reminders are a first step in a process of evidence-based care but are not the complete process

At the same time, participants made clear that they valued certain aspects of the technology. They valued the ability to make referrals efficiently, and to provide education to other clinicians through templated forms. They valued reminders about important clinical tasks, despite their concerns that responding to reminders took up precious time.

In summary, our results underscore the complexities of EHR implementation. Participants described a tension between the value added to their work by the EHR, and barriers to its enthusiastic adoption. The chief barrier was anxiety about technology detracting from the patient-provider relationship, either subtracting from the time available or altering the interpersonal dynamic. Designing the EHR to minimize intrusiveness in the patient-provider relationship may reduce this barrier to implementation. For example, McGrath and colleagues [35] and Frankel and colleagues [36] found that the physical positioning of the computer within the exam room affected nonverbal communication, such as eye contact, between providers and patients. Frankel and colleagues, however, found that use of the EHR seemed to improve good communication skills and worsen already poor skills [36]; their findings provide another example of the complexity of the role of the EHR in clinical situations. Given the documented importance of provider-patient interaction [37-39], more research into the role of EHRs in facilitating this interaction is necessary. Likewise, streamlining data entry as much as possible may improve implementation among providers who 'just want to take care of the patients.'

Prior research has found differences in interactions with the EHR by profession [40,41]. Our research was not designed to explore this question, but some participants expressed opinions about how different professionals should use the EHR (for example, stating that 'clerks should be doing [clinical reminders]'). Future EHR implementation projects may benefit from careful exploration of which tasks are appropriate for different professions.

Finally, we suggest that, throughout the design and implementation process, administrators obtain data from end-users, not only about barriers, but also about what they value in the EHR. Some such data may be obtained through formal usability testing prior to implementation. It is probably just as important to conduct ongoing assessments to detect concerns during and after the initial implementation period. For example, the concern raised by one respondent about inappropriate information being included in EHR notes might not have arisen during initial usability testing. Knowledge about what is valued will help EHR designers combine what end-users want with what administrators need; this process may also facilitate EHR adoption. The results of this study and future studies may provide information that can be used to encourage EHR adoption. For example, administrators may want to describe the benefits perceived by previous users when implementing a new EHR. The PDSA cycle and the informatics-specific ITSA cycle both describe a process in which real-world experience informs system design. Successful implementation of an EHR may require such a process of ongoing evaluation, in which feedback from end-users helps EHR designers maximize the valued attributes of the system and address the barriers they encounter. As one respondent pointed out, addressing barriers may be 'a system issue' in which information technology personnel, clinicians, and administrators must collaborate in order to address barriers and maximize the value of the EMR.

### Recommendations

In summary, we recommend that software designers conduct ongoing usability assessment to detect end-user's frustrations with the EHR, and work to minimize these problems. For example, if clinicians repeatedly report that 'checklists' interfere with the patient-provider relationship, administrators might delegate more of the routine reminders to support staff. We equally recommend that software designers find out what end-users most like about the EHR and work to enhance these features. For example, if clinicians report that they appreciate being able to communicate with the rest of the care team through the EHR, designers might invest effort in making this communication process as easy and informative as possible. Please refer to Table 5 for a summary of recommendations for implementation.

**Table 5:** Lessons and recommendations for EHR implementation

Barriers	Recommended facilitators
Concerns about time	Emphasize efficiency, potential time savings (for example, asynchronous communication, templated notes if appropriate)

Concerns about effect on relationship with patients	1. Physical positioning of computers to minimize disruption of eye contact, *et al.* 2. Emphasize positive effects on relationship (reminders opening up important conversations, *et al.*)

Concerns about effect on relationship with colleagues	Emphasize efficiency, quality of care

### Limitations

Because we base our findings on experiences within the VA, they are most applicable to large managed care systems and may be less applicable to small healthcare organizations and private practices. We did not specifically design this study to examine EHR implementation. Rather, participants discussed the EHR in the course of interviews designed to study a depression QI project. Therefore, our participants' comments may be most applicable to the use of informatics in a QI context.

## Summary

VA staff members valued the efficiency and support for quality of care offered by the EHR. However, they expressed serious concerns about the EHR's potential interference with the provider-patient relationship, and were keenly aware of the time cost of using the EHR. We suggest that EHR designers obtain ongoing feedback from end-users. Learning what barriers exist is essential to addressing them. Likewise, learning which EHR attributes are most valued -- and why -- will allow designers to enhance these features, potentially making the EHR more appealing to end-users.

## Competing interests

The authors declare that they have no competing interests.

## Authors' contributions

LB conducted data analysis and drafted the manuscript. CS conducted data analysis and helped to draft the manuscript. LP participated in study design, data collection and analysis, and helped to draft the manuscript. EY participated in study design and data collection, and helped to draft the manuscript. JK led the study, participated in study design and data collection, and helped to draft the manuscript. All authors read and approved the final manuscript.
